# Retention in Care and Outpatient Costs for Children Receiving Antiretroviral Therapy in Zambia: A Retrospective Cohort Analysis

**DOI:** 10.1371/journal.pone.0067910

**Published:** 2013-06-28

**Authors:** Callie A. Scott, Hari Iyer, Deophine Lembela Bwalya, Kelly McCoy, Gesine Meyer-Rath, Crispin Moyo, Carolyn Bolton-Moore, Bruce Larson, Sydney Rosen

**Affiliations:** 1 Center for Global Health and Development, Boston University, Boston, Massachusetts, United States of America; 2 Zambia Center for Applied Health Research and Development, Lusaka, Zambia; 3 Health Economics and Epidemiology Research Office, Wits Health Consortium, Faculty of Health Sciences, University of the Witwatersrand, Johannesburg, South Africa; 4 Zambian Ministry of Health, Lusaka, Zambia; 5 Department of Obstetrics & Gynecology, University of North Carolina at Chapel Hill, Chapel Hill, North Carolina, United States of America; 6 Centre for Infectious Disease Research in Zambia, Lusaka, Zambia; 7 Department of International Health, School of Public Health, Boston University, Boston, Massachusetts, United States of America; Indiana University, United States of America

## Abstract

**Background:**

There are few published estimates of the cost of pediatric antiretroviral therapy (ART) in Africa. Our objective was to estimate the outpatient cost of providing ART to children remaining in care at six public sector clinics in Zambia during the first three years after ART initiation, stratified by service delivery site and time on treatment.

**Methods:**

Data on resource utilization (drugs, diagnostics, outpatient visits, fixed costs) and treatment outcomes (in care, died, lost to follow up) were extracted from medical records for 1,334 children at six sites who initiated ART at <15 years of age between 2006 and 2011. Fixed and variable unit costs (reported in 2011 USD) were estimated from the provider’s perspective using site level data.

**Results:**

Median age at ART initiation was 4.0 years; median CD4 percentage was 14%. One year after ART initiation, 73% of patients remained in care, ranging from 60% to 91% depending on site. The average annual outpatient cost per patient remaining in care was $209 (95% CI, $199–$219), ranging from $116 (95% CI, $107–$126) to $516 (95% CI, $499–$533) depending on site. Average annual costs decreased as time on treatment increased. Antiretroviral drugs were the largest component of all outpatient costs (>50%) at four sites. At the two remaining sites, outpatient visits and fixed costs together accounted for >50% of outpatient costs. The distribution of costs is slightly skewed, with median costs 3% to 13% lower than average costs during the first year after ART initiation depending on site.

**Conclusions:**

Outpatient costs for children initiating ART in Zambia are low and comparable to reported outpatient costs for adults. Outpatient costs and retention in care vary widely by site, suggesting opportunities for efficiency gains. Taking advantage of such opportunities will help ensure that targets for pediatric treatment coverage can be met.

## Introduction

At the end of 2010, 456,000 children in low- and middle-income countries were reported to be on antiretroviral therapy (ART) for HIV/AIDS, representing only 23% of children in need of ART [1]. Although pediatric ART coverage lags adult coverage substantially, an ever-increasing proportion of HIV-infected children are initiating ART each year, of whom some 85% live in sub-Saharan Africa [1]. Treatment outcomes for these children vary widely but are generally encouraging [2]. In Zambia, a low-middle income country in southern Africa where 25,388 children were reported to be on ART and another 72,612 children were reported to be in need of ART in 2010 [1], several papers have reported on good clinical, immunologic, and virologic outcomes for children through two years after ART initiation [3], [4].

Recent research on the outcomes of pediatric ART in Africa has not been matched by research on the costs of treatment. A review of the costs of HIV treatment in developing countries published in 2011 found one published study that reported pediatric HIV costs and we are aware of only two studies published since then [5], [6], [7], [8]. No published estimates exist for Zambia. For Ministries of Health, funding agencies, and others responsible for provision of medical services and ensuring sufficient resources to achieve national and international HIV response goals, there is effectively no information available about this important component of national treatment programs.

To contribute to efforts to deliver HIV services more efficiently and ensure that sufficient resources are available for treatment programs to be sustained, we estimated the outpatient costs of providing ART to children attending six public sector clinics in Zambia during the first three years after ART initiation, stratified by service delivery site and time on treatment.

## Methods

### Ethics Statement

The Boston University Medical Center Institutional Review Board and the University of Zambia Research Ethics Committee provided ethical approval of the study (protocol numbers H-28104 and 008-04-09). A waiver of informed consent was granted by both committees because the study was a retrospective review of routinely collected information from patient medical records.

### Analytic Overview

We used previously published methods to estimate average annual outpatient costs per patient for the total cohort and for the subset remaining in care [8], [9], [10]. We used retrospective cohort data from outpatient medical records on patient outcomes and resource utilization and site-level data on unit costs. Resources utilized included antiretroviral (ARV) drugs, laboratory tests, outpatient visits, and fixed costs (e.g., buildings and infrastructure, equipment, supplies). Costs were calculated from the provider’s perspective in 2011 US dollars.

### Study Sites

We purposively selected six study sites intended to represent specific models or settings for pediatric ART delivery in Zambia (Table 1). Sites included a health center in Lusaka Province (site 1), a second-level general hospital in Western Province (site 2), a pediatric center of excellence operating within a second-level general hospital in Southern Province (site 3), a second-level general hospital in Copperbelt Province (site 4), a second-level mission hospital in Southern Province (site 5), and a first-level district hospital in Southern Province (site 6). All sites were public sector clinics funded primarily by the Government of Zambia and providing ART services to patients free of charge. The number of active adult and pediatric patients enrolled in the ART program at each site in 2010 ranged from 827 at site 3, which sees pediatric patients only, to 6,640 at site 2.

**Table 1:** Study site and cohort characteristics in an analysis of the costs and outcomes of pediatric ART in Zambia.

	Site 1	Site 2	Site 3	Site 4	Site 5	Site 6	All sites
*Site characteristics*							
Province	Lusaka	Western	Southern	Copperbelt	Southern	Southern	–
Description	Health center	Second-level general hospital	Pediatric center of excellence	Second-level general hospital	Second-level mission hospital	First-level district hospital	–
Setting	Urban	Rural	Urban	Urban	Rural	Peri-urban	–
Number of active patients enrolled in ART program, 2010	6,084	6,640	827	2,994	4,202	4,390	–
*Cohort characteristics at ART initiation*							
Calendar years of ART initiation	2006–2009	2006–2009	2008–2009	2006–2010	2006–2011	2006–2010	2006–2011
Median age at ART initiation, years [IQR]	5.4 [2.3–9.4]	2.6 [1.3–6.4]	1.9 [1.0–6.1]	5.1[1.9–8.6]	4.4 [1.9–8.5]	5.6 [2.2–8.5]	4.0 [1.7–7.9]
Median CD4 at ART initiation, % [IQR]	12 [8]–[16]	n.a.	16 [10–22]	13 [8–21]	14 [9–20]	11 [6–20]	14 [9–20]
Regimen at ART initiation, % of patients							
d4T+3TC+NVP or EFV	60	77	75	75	70	92	75
AZT+3TC+NVP or EFV	40	15	6	22	29	5	19
Other regimen	0	9	20^a^	4	1	2	6
*Total sample size by duration of follow up*							
12 months	187	262	232	166	275	212	1,334
24 months	126	185	120	120	195	154	900
36 months	120	120	0	0	120	89	449

3TC: lamivudine; ART: antiretroviral therapy; AZT: zidovudine; d4T: stavudine; EFV: efavirenz; IQR: interquartile range; NVP: nevirapine.

a The majority of patients initiating ART on an “other” regimen at site 3 initiated with d4T+3TC+ABC or AZT+3TC+ABC because they had a confirmed or presumptive diagnosis of TB at the time of initiation.

### Sample Selection

At each study site, up to three retrospective cohorts of 120 children each who initiated ART at the site at least 12, 24, or 36 months prior to data collection were selected consecutively from clinic registers and enrolled in the study. Due to low patient volume at the study sites, the resulting cohorts overlapped, creating a single cohort of study subjects who initiated ART at least 12 months prior to data collection at each site. Data for each subject was censored at 12, 24, or 36 months after ART initiation, allowing between 12 and 36 months of follow up for each study subject.

Eligible study subjects were children between 0 and 14 years of age at ART initiation who were not known to have transferred to another clinic during the study follow up period. Medical eligibility for ART initiation followed Zambian national guidelines prevailing during the study period and included initiation irrespective of CD4 or WHO clinical stage for all children under 12 months of age, initiation irrespective of CD4 for children ≥12 months of age with WHO clinical stage 3 or 4, and initiation according to CD4 cell count or total lymphocyte count thresholds for children ≥12 months of age with WHO clinical stage 1 or 2 [11].

### Data Collection

Data on resources used during the first 12, 24, or 36 months following ART initiation were obtained from each study subject’s medical record. With the exception of non-ARV drugs, all resources used by the provider to provide outpatient care to study subjects were included, even if the resource cost was borne by another entity (e.g., by an external funder). Non-ARV drugs were excluded because of the poor quality of information recorded in the study subjects’ medical records. Fixed and variable unit costs for resources utilized were estimated from site-level financial records, interviews with site managers, national drug price lists, and market prices.

### Classification of Patient Outcomes

Each study subject was assigned to an outcome category at the end of 12 months. Those with sufficient follow up were also assigned to outcome categories after 24 and 36 months on ART. Outcomes were defined on the basis of subject status at the study site and included *in care*, *known to have died*, and *lost to follow up*.

Subjects were classified as *known to have died* if a confirmation of death was noted in their medical record before the 12-, 24-, or 36-month study endpoint. Subjects who were three or more months late for their last scheduled consultation or medication pickup before the 12-, 24-, or 36-month study endpoint but had no confirmation of death in their medical record were classified as *lost to follow up*. Subjects not classified as known to have died or lost to follow up were classified as *in care*.

### Cost Estimates

Fixed and variable unit costs used in the analysis included costs incurred at the site reported in Zambian Kwacha (ZMK) adjusted to 2011 levels using the country’s consumer price index [12]. All costs were converted to US dollars at a rate of 4,861 ZMK/$, the average exchange rate for 2011 [13].

Fixed costs were defined as costs for resources used to treat study subjects that could not be attributed directly to an individual subject’s care, such as buildings, equipment, and support staff in the ART clinic. For equipment and buildings, upfront investment costs were estimated using a replacement cost approach. These costs were annualized using a 3% discount rate and an estimated working life (50 years for buildings, 5 years for equipment) [14]. The annual cost of support staff employed in the ART clinic at the study sites during the study period was based on 2011 salaries and allowances. The total annual fixed cost for each site was estimated and then divided by the total number of active patients at the site during each year to produce an average fixed cost per patient-year in care.

Variable costs were defined as costs for resources used to treat study subjects that could be attributed directly to an individual subject or visit, such as ARV drugs, laboratory tests, and provider time for clinic visits. ARV drug costs were estimated as the average per unit cost for all units of a particular drug purchased for the Zambia national HIV program in 2011, or during the most recent year available if no units of a particular drug were purchased in 2011, as reported by the Global Price Reporting Mechanism [15]. Data on ART regimen dispensed and patient weight at each visit were used to determine the appropriate drug formulation (fixed dose tablet, single dose tablet, or syrup) and dose for each ARV drug dispensed [11]. Laboratory test costs were estimated as the sum of unit costs for reagents, consumables, equipment, labor, and space. Costs of reagents and consumables were estimated from standard Zambian Ministry of Health per package costs [16]. Annual laboratory equipment, labor, and space costs were divided by the total number of laboratory tests performed per year to estimate a per test cost at each site. Provider time was valued on a per visit basis. The cost per visit was estimated by dividing the total cost of staff time for each type of provider conducting patient consultations, valued at 2011 salaries and allowances, by the total number of patient consultations with each provider type per year.

Costs for outpatient care above the level of the service delivery site (e.g., government costs of oversight or training) and costs to the patients (e.g., clinic fees, transport, caregiver time associated with child visits) were excluded..

### Data Analysis

Data were analyzed in Stata version 11. Average annual costs per patient for the total sample were calculated at each time point –12, 24, or 36 months after ART initiation – by dividing total costs for the first one, two, or three years on ART for all patients by the total number of patients in the cohort and then dividing by one, two, or three years. Average annual costs per patient for the subset of the sample remaining in care were calculated by dividing total costs for the subset of the sample remaining in care by the total number of patients remaining in care at each time point. Average annual costs to produce a patient remaining in care at 12, 24, or 36 months after ART initiation, referred to as production costs, were calculated by dividing the average cost for all patients in the total sample over the first one, two, or three years on ART by the proportion of patients remaining in care at each time point and then dividing by one, two, or three years. All average and production costs are reported with 95% confidence intervals. Confidence intervals around the average cost to produce a patient remaining in care at each site, which is a ratio of two random variables, were estimated using bootstrapping methods.

For the subset of patients remaining in care 12 months after initiating ART, we estimated a linear regression model of the natural log of the total cost per patient for the first year on ART against study site, calendar year of ART initiation, age at initiation, nucleoside reverse transcriptase inhibitor (NRTI) combination at initiation, and non-nucleoside reverse transcriptase inhibitor (NNRTI) or protease inhibitor (PI) at initiation. We used the *reg* command in Stata with robust standard errors. A p-value of less than 0.05 was regarded as significant.

## Results

### Cohort Characteristics

A total of 1,334 patients initiating ART at the six study sites between 2006 and 2011 were included in the retrospective cohort (Table 1). All 1,334 patients contributed data to the 12-month study endpoint; 900 and 449 patients contributed data to the 24- and 36-month study endpoints respectively. The maximum duration of follow up at sites 3 and 4 was only 24 months due to incomplete medical records (site 3) and insufficient numbers (site 4) for patients initiated on ART at least 36 months prior to data collection.

Median age at ART initiation was 4.0 years; median CD4 percentage was 14%. A majority of patients at all six sites initiated on a regimen containing stavudine, lamivudine, and either nevirapine or efavirenz, with most of the rest substituting zidovudine for stavudine. Few patients switched from a first-line to a second-line regimen, defined as a switch from an NNRTI-based regimen to a PI-based regimen or vice versa, during the study follow up period (1% switched during the first year after ART initiation and less than 1% switched during each of the second and third years after ART initiation).

### Patient Outcomes

One year after ART initiation, 73% of patients remained in care, 5% were known to have died, and 22% were lost to follow up (Table 2). The proportion of patients remaining in care ranged from 60% at site 2 to 91% at site 5. Three years after ART initiation, 67% of patients remained in care, ranging from 55% at site 2 to 90% at site 5.

**Table 2:** Patient outcomes at six ART treatment sites in Zambia by site and time on treatment.

Patient outcome, n (%)	Site 1	Site 2	Site 3	Site 4	Site 5	Site 6	All sites
*12 months after ART initiation*							
In care	147 (79)	156 (60)	171 (74)	116 (70)	250 (91)	137 (65)	977 (73)
Known to have died	28 (15)	16 (6)	3 (1)	8 (5)	1 (0)	11 (5)	67 (5)
Lost to follow up	12 (6)	90 (34)	58 (25)	42 (25)	24 (9)	64 (30)	290 (22)
*24 months after ART initiation*							
In care	87 (69)	107 (58)	82 (68)	67 (56)	185 (95)	92 (60)	620 (69)
Known to have died	21 (17)	14 (8)	2 (2)	7 (6)	0 (0)	9 (6)	53 (6)
Lost to follow up	18 (14)	64 (35)	36 (30)	46 (38)	10 (5)	53 (34)	227 (25)
*36 months after ART initiation*							
In care	76 (63)	66 (55)	n.a.	n.a.	108 (90)	50 (56)	300 (67)
Known to have died	21 (18)	11 (9)	n.a.	n.a.	0 (0)	3 (3)	35 (8)
Lost to follow up	23 (19)	43 (36)	n.a.	n.a.	12 (10)	36 (40)	114 (25)

ART: antiretroviral therapy.

### Cost Per Patient

For the first year after ART initiation, the average annual cost per patient for the total sample was $169 (95% CI, $161–$178), ranging from $81 (95% CI, $73–$89) at site 2 to $433 (95% CI, $408–$458) at site 3 (Table 3). The average annual cost per patient for the subset of the sample remaining in care at 12 months was $209 (95% CI, $199–$219), ranging from $116 (95% CI, $107–$126) at site 2 to $516 (95% CI, $499–$533) at site 3. The distribution of costs was slightly skewed at all six sites with median costs 3% to 13% lower than average costs per patient for the subset of the sample remaining in care during the first year after ART initiation.

**Table 3:** Average annual costs at six ART treatment sites in Zambia by site and time on treatment.

Average annual cost, 2011 USD (95% CI)	Site 1	Site 2	Site 3	Site 4	Site 5	Site 6	All sites
*12 months after ART initiation*							
Average annual cost per patient for total sample	123 (114–131)	81 (73–89)	433 (408–458)	114 (102–126)	156 (148–165)	92 (84–100)	169 (161–178)
Average annual cost per patient for subset of sample remaining in care	148 (142–154)	116 (107–126)	516 (499–533)	151 (140–163)	165 (157–174)	125 (119–132)	209 (199–219)
*24 months after ART initiation*							
Average annual cost per patient for total sample	114 (102–126)	68 (60–75)	372 (343–401)	91 (78–104)	155 (148–163)	84 (74–94)	140 (131–148)
Average annual cost per patient for subset of sample remaining in care	152 (144–161)	105 (98–111)	457 (437–477)	142 (129–155)	158 (152–165)	126 (119–132)	181 (172–191)
*36 months after ART initiation*							
Average annual cost per patient for total sample	108 (95–121)	62 (52–71)	n.a.	n.a.	150 (142–158)	80 (67–93)	101 (95–107)
Average annual cost per patient for subset of sample remaining in care	149 (139–160)	102 (95–109)	n.a.	n.a.	155 (147–164)	125 (115–134)	137 (132–142)

ART: antiretroviral therapy; CI: confidence interval; USD: United States dollar.

At five of the six sites, average annual costs per patient for the subset of the sample remaining in care two years after ART initiation were lower (0–11% less) than average annual costs per patient for the subset remaining in care during the first year after ART initiation. Average annual costs per patient for the subset of the sample remaining in care three years after ART initiation were essentially the same as average annual costs per patient for the subset remaining in care during the first two years after ART initiation at all sites.

Table 4 presents results from a regression of the natural log of the total cost per patient for the first year on ART against site and patient-specific variables. ART initiation in a later calendar year, initiation at a younger age, initiation on a stavudine- and lamivudine- containing regimen (versus a regimen containing zidovudine and lamivudine or other NRTI combinations), and initiation on a nevirapine-containing regimen (versus a regimen containing efavirenz or other NNRTI or a PI), were all significantly associated with lower total costs per patient during the first year on ART.

**Table 4:** Ordinary least squares regression of the natural log of total per patient costs for the first year on ART against study site and patient-specific characteristics^a^.

Variable	Coefficient^b^	t	P-value	95% CI
*Dummy for site* c				
Site 2	−0.323	−9.38	<0.001	(−0.391, −0.256)
Site 3	1.326	36.59	<0.001	(1.255, 1.397)
Site 4	0.002	0.04	0.97	(−0.082, 0.085)
Site 5	0.218	6.58	<0.001	(0.153, 0.284)
Site 6	−0.057	−1.81	0.07	(−0.118, 0.005)
*Dummy for ART initiation year* d				
2007	0.071	1.79	0.07	(−0.007, 0.150)
2008	−0.062	−1.51	0.13	(−0.142, 0.018)
2009	−0.167	−4.20	<0.001	(−0.244, −0.089)
2010	−0.289	−5.57	<0.001	(−0.391, −0.187)
2011	−0.280	−3.68	<0.001	(−0.429, −0.131)
*Age at initiation in years*	0.012	4.48	<0.001	(0.007, 0.017)
*Dummy for NRTI combination at ART initiation* e				
AZT+3TC	0.178	6.78	<0.001	(0.126, 0.229)
Other NRTI combination	0.847	12.53	<0.001	(0.714, 0.979)
*Dummy for NNRTI/PI at ART initiation* f				
EFV	0.232	7.50	<0.001	(0.171, 0.292)
Other NNRTI or PI	0.017	0.69	0.49	(−0.031, 0.065)
*Intercept*	4.829	124.46	<0.001	(4.753, 4.906)

3TC: lamivudine; ABC: abacavir; ART: antiretroviral therapy; AZT: zidovudine; CI: confidence interval; d4T: stavudine; EFV: efavirenz; FTC: emtricitabine; IDV: indinavir; LPV/r: ritonavir-boosted lopinavir; NFV: nelfinavir; NNRTI: non-nucleoside reverse transcriptase inhibitor; NRTI: nucleoside reverse transcriptase inhibitor; NVP: nevirapine; PI: protease inhibitor; TDF: tenofovir.

a Analysis includes 945 patients in the subset of the sample remaining in care 12 months after ART initiation. Analysis excludes 32 patients in the subset of the sample remaining in care 12 months after ART initiation who initiated ART with a triple NRTI regimen because they had a confirmed or presumptive diagnosis of TB.

b For interpretation, the coefficient multiplied by 100 represents the percentage change in total costs for a one unit change in the explanatory variable.

c Reference is site 1.

d Reference is initiation in 2006.

e Reference is initiation on a regimen containing d4T+3TC.

f Reference is initiation on a regimen containing NVP. The “other” category includes LPV/r, IDV, and NFV.

### Cost Breakdown

Antiretroviral drugs were the largest cost component at 4 of 6 sites, comprising between 52% (site 6) and 70% (site 2) of all outpatient costs per patient for the subset of the sample remaining in care during the first year after ART initiation (Table 5). At these sites, outpatient visits were the next largest cost component, comprising between 17% (site 2) and 31% (site 6) of all outpatient costs, while fixed costs and laboratory tests were a smaller share of costs per patient. At sites 3 and 5, outpatient visits and fixed costs together made up the majority of all outpatient costs per patient for the subset of the sample remaining in care during the first year after ART initiation.

**Table 5:** Breakdown of per patient costs by cost item during the first year on treatment at six ART treatment sites in Zambia.

Average cost per patient for subset of sample remaining in care 12 months after initiating ART, 2011 USD (% of total)	Site 1	Site 2	Site 3	Site 4	Site 5	Site 6	All sites
ARV drugs	82 (55)	82 (70)	101 (20)	80 (53)	64 (39)	65 (52)	78 (37)
Laboratory tests	11 (7)	8 (7)	7 (1)	23 (15)	14 (8)	13 (10)	12 (6)
Outpatient visits^a^	42 (28)	19 (17)	241 (47)	35 (23)	66 (40)	39 (31)	78 (37)
Fixed costs^b^	13 (9)	7 (6)	167 (32)	13 (9)	22 (13)	9 (7)	41 (20)
Total	148 (100)	116 (100)	516 (100)	151 (100)	165 (100)	125 (100)	209 (100)

ART: antiretroviral therapy; ARV: antiretroviral; USD: United States dollar.

a Outpatient visits include the cost of staff time for staff who conduct patient consultations.

b Fixed costs include the cost of buildings and infrastructure, equipment, supplies, vehicles, and staff time for staff in ART clinic who do not conduct patient consultations.

### Production Cost

One year after ART initiation, the annual cost to produce a patient remaining in care was $231 (95% CI, $220–$242), ranging from $136 (95% CI, $126–$147) at site 2 to $588 (95% CI, $560–$622) at site 3 (Table 6). Two years after ART initiation, the annual cost to produce a patient remaining in care was $203 (95% CI, $193–$214), ranging from $117 (95% CI, $110–$125) at site 2 to $545 (95% CI, $506–$595) at site 3.

**Table 6:** Average annual cost to produce a patient in care at six ART treatment sites in Zambia by site and time on treatment.

Average annual cost, 2011 USD (95% CI)	Site 1		Site 2	Site 3	Site 4	Site 5	Site 6	All sites
*12 months after ART initiation*								
Average annual cost per patient for total sample	123 (114–131)		81 (73–89)	433 (408–458)	114 (102–126)	156 (148–165)	92 (84–100)	169 (161–178)
% of total sample remaining in care	79		60	74	70	91	65	73
Average annual cost to produce a patient remaining in care	156 (149–164)		136 (126–147)	588 (560–622)	164 (150–177)	172 (163–181)	142 (134–153)	231 (220–242)
*24 months after ART initiation*								
Average annual cost per patient for total sample	114 (102–126)		68 (60–75)	372 (343–401)	91 (78–104)	155 (148–163)	84 (74–94)	140 (131–148)
% of total sample remaining in care	69		58	68	56	95	60	69
Average annual cost to produce a patient remaining in care	165 (155–176)		117 (110–125)	545 (506–595)	164 (148–183)	164 (156–172)	141 (132–152)	203 (193–214)
*36 months after ART initiation*								
Average annual cost per patient for total sample		108 (95–121)	62 (52–71)	n.a.	n.a.	150 (142–158)	80 (67–93)	101 (95–107)
% of total sample remaining in care		63	55	n.a.	n.a.	90	56	67
Average annual cost to produce a patient remaining in care		171 (155–187)	111 (104–121)	n.a.	n.a.	167 (157–178)	142 (129–159)	151 (145–159)

ART: antiretroviral therapy; CI: bootstrapped confidence interval; USD: United States dollar.

## Discussion

The lack of information available about the cost of providing ART to children in low-income African countries limits the capacity of policy makers and funding agencies to achieve global targets for pediatric treatment coverage. To help fill this gap, we estimated the average annual outpatient cost of providing ART to children remaining in care at six public sector clinics in Zambia during the first three years after treatment initiation, stratified by service delivery site and time on treatment. One year after ART initiation, 73% of patients remained in care, ranging from 60% to 91% depending on study site. The average annual outpatient cost per patient for the subset of the sample remaining in care was $209 (95% CI, $199–$219), ranging from $116 (95% CI, $107–$126) to $516 (95% CI, $499–$533) depending on study site. This average annual outpatient cost per patient decreased as time on treatment increased at most sites. The distribution of costs was skewed, with median costs 3% to 13% lower than average costs depending on the site. ARV drugs were the largest cost component, making up more than 50% of all outpatient costs at four of the six sites. Outpatient visits and fixed costs made up the majority of outpatient costs at the remaining two sites.

Some sites had higher proportions of patients retained in care than others. While some of this variation may be due to differences in patient characteristics, such as age at initiation or CD4 percentage at presentation, and other differences in the underlying patient populations across sites, some may also be due to differences in quality of care. To the extent that variation in patient outcomes was driven by quality of care, the exceptional patient outcomes at site 5, where 90% of patients remained in care three years after initiating ART, suggest that there may be potential to improve retention in care, and thereby increase efficiency, at other sites.

Some sites also had higher costs than others and patient outcomes were not necessarily better at sites with higher costs. Variation in costs across sites may be due to factors including, but not limited to, differences in the frequency of outpatient visits and diagnostic tests performed, choice of ART regimen, staffing levels, and patient volume. This variation again suggests opportunities to improve efficiency. For example, at site 3, the dedicated pediatric clinic and by far the most expensive model of care, there may be potential to reduce costs and increase efficiency by moving to a more integrated care model – by integrating adult and pediatric ART services or integrating pediatric ART and other pediatric outpatient services – where a similar level of staffing and fixed costs could be used to serve a larger number of patients.

Some individuals also incurred markedly higher costs than others. The skewed cost distribution at the study sites suggests that a small number of high cost patients may be driving up the average cost per patient remaining in care at these sites. We found that initiation at a younger age, initiation on a stavudine- and lamivudine-containing regimen (versus a regimen containing other NRTI combinations), and initiation on a nevirapine-containing regimen (versus a regimen containing another NNRTI or PI) were all significantly associated with lower per patient costs. These associations suggest that there may be opportunities for efficiency gains through earlier identification and linkage to care of HIV-infected children and increased standardization of ART regimens.

The costs we estimated for pediatric ART in Zambia are comparable to estimates of the costs of adult ART in Zambia. Bratt et al. estimated annual costs ranging from $278 to $523 (in 2008 US dollars) per patient for the first year on ART [17]. Tagar et al. estimated an average cost of $278 per ART patient per year in 2010–2011 [18]. Marseille et al. estimated a cost of $428 per person-year of ART in on-site costs (in 2010 US dollars), and an additional $210 per person-year of ART in off-site costs, for the average facility in their sample [19].

There are only two other recently published studies reporting outpatient costs of pediatric ART [6]. Menzies et al. estimated costs for pediatric patients in four countries, including three in Africa (Ethiopia, Nigeria, and Uganda) [6]. Average annual costs per newly initiated pediatric ART patient ranged from $374 in Uganda to $1,771 in Nigeria (in 2009 US dollars). Average annual costs per established patient ranged from $454 in Uganda to $1,564 in Nigeria. While not strictly comparable to our results – this study included costs of administration and management above the site level – the estimates from other countries suggest that there is a large amount of variation in pediatric treatment costs between countries as well as between sites. Meyer-Rath et al. estimated the cost of providing pediatric ART during the first two years after ART initiation at two sites in South Africa [8]. Costs were estimated using methods comparable to those in our study and ranged from $678 to $830 per patient remaining in care during the first year and $717 to $782 per patient remaining in care during the second year (in 2009 US dollars). These costs for pediatric ART in South Africa are substantially higher than the costs we estimated for pediatric ART in Zambia. This difference can be explained, at least in part, by higher staffing and fixed costs in South Africa, which is an upper-middle income country, higher drug costs, and higher laboratory costs due to the use of viral load monitoring which is not routinely available in Zambia.

Our study has several limitations. First, results are from six purposively selected sites. Sites were chosen to capture variation in location, size, and delivery model, rather than to represent the ART treatment program in Zambia as a whole. Because sites were not selected randomly, average costs and retention rates for our six-site sample should not be taken as an average for the country or as an average for any particular type of site. Further, because we have only six sites in our sample, we cannot attribute variation in costs of care between sites to specific site-level characteristics. Second, patient outcomes in this analysis are limited to what could be ascertained from a retrospective review of medical records. For patients no longer attending the study clinic, we could not always distinguish between those who had transferred to another clinic, died, or been lost to follow up due to incomplete records. Third, we excluded patients known to have transferred to another site during the study follow up period. These patients may differ systematically from the study population. Fourth, results are for average, not marginal, costs. The cost of scaling up any of these six programs or of opening a new ART clinic based on a similar model in a similar setting may differ from our estimates. Fifth, we excluded costs of non-ARV drugs, although such costs, when estimated, are typically a small share of outpatient costs [8], [9]. Sixth, costs of inpatient care, costs incurred before a patient initiates ART, costs incurred by the patient, and costs for program management above the facility level were excluded from the analysis, leading to an underestimate of the total cost to Zambia of providing pediatric ART. Finally, results reflect the costs and retention in care for patients who, for the most part, initiated ART prior to the adoption of early infant diagnosis and treatment guidelines in Zambia in 2010.

In conclusion, outpatient costs for children initiating ART in Zambia are low and are comparable to reported outpatient costs for adults initiating ART in Zambia. Outpatient costs and retention in care vary widely by site. Exploring such variation will provide insight into opportunities for efficiency gains through improvements in retention in care and decreases in service delivery costs for patients who remain in care. Taking advantage of such opportunities for efficiency gains will help ensure that targets for pediatric treatment coverage can be met.

## References

[1] World Health Organization (2011) Global HIV/AIDS response: epidemic update and health sector progress towards universal access: progress report 2011. Available at: http://whqlibdoc.who.int/publications/2011/9789241502986_eng.pdf. Accessed: 3 September 2012.

[2] Ciaranello AL, Chang Y, Margulis AV, Bernstein A, Bassett IV, et al (2009) Effectiveness of pediatric antiretroviral therapy in resource-limited settings: a systematic review and meta-analysis. Clin Infect Dis 49: 1915–1927. 19916798 10.1086/648079 PMC2787758

[3] Bolton-Moore C, Mubiana-Mbewe M, Cantrell RA, Chintu N, Stringer EM, et al (2007) Clinical outcomes and CD4 cell response in children receiving antiretroviral therapy at primary health care facilities in Zambia. JAMA 298: 1888–1899. 17954540 10.1001/jama.298.16.1888

[4] van Dijk JH, Sutcliffe CG, Munsanje B, Sinywimaanzi P, Hamangaba F, et al (2011) HIV-infected children in rural Zambia achieve good immunologic and virologic outcomes two years after initiating antiretroviral therapy. PLoS One 6: e19006. 21552521 10.1371/journal.pone.0019006 PMC3084269

[5] Galarraga O, Wirtz VJ, Figueroa-Lara A, Santa-Ana-Tellez Y, Coulibaly I, et al (2011) Unit costs for delivery of antiretroviral treatment and prevention of mother-to-child transmission of HIV: a systematic review for low- and middle-income countries. Pharmacoeconomics 29: 579–599. 21671687 10.2165/11586120-000000000-00000 PMC3833352

[6] Menzies NA, Berruti AA, Berzon R, Filler S, Ferris R, et al (2011) The cost of providing comprehensive HIV treatment in PEPFAR-supported programs. AIDS 25: 1753–1760. 21412127 10.1097/QAD.0b013e3283463eec PMC3225224

[7] Marques HH, Couttolenc BF, Latorre Mdo R, Aquino MZ, Aveiro MI, et al (2007) Costs of care provided in a university hospital for children exposed to or infected with the HIV/AIDS. Cad Saude Publica 23 Suppl 3S402–413. 17992346 10.1590/s0102-311x2007001500008

[8] Meyer-Rath G, Brennan A, Long L, Ndibongo B, Technau K, et al (2013) Cost and outcomes of paediatric antiretroviral treatment in South Africa. AIDS 27: 243–250. 23014517 10.1097/QAD.0b013e32835a5b92

[9] Rosen S, Long L, Sanne I (2008) The outcomes and outpatient costs of different models of antiretroviral treatment delivery in South Africa. Trop Med Int Health 13: 1005–1015. 18631314 10.1111/j.1365-3156.2008.02114.x

[10] Long L, Fox M, Sanne I, Rosen S (2010) The high cost of second-line antiretroviral therapy for HIV/AIDS in South Africa. AIDS 24: 915–919. 20042849 10.1097/QAD.0b013e3283360976

[11] Government of the Republic of Zambia, Ministry of Health (2007) Zambian guidelines for antiretroviral therapy of HIV infection in infants and children: towards universal access, 2007. Available: http://www.who.int/hiv/amds/zambia_paediatric_guidelines_2007.pdf. Accessed 3 September 2012.

[12] International Monetary Fund (2012) World Economic Outlook Database, April 2012. Available: http://www.imf.org/external/pubs/ft/weo/2012/01/weodata/index.aspx. Accessed 3 September 2012.

[13] Oanda Corporation (2012) Historical exchange rates: daily midpoint rates: January 1, 2011 to December 31, 2011. Available: www.oanda.com/currency/historical-rates/. Accessed 2 March 2012.

[14] Weinstein MC, Siegel JE, Gold MR, Kamlet MS, Russell LB (1996) Recommendations of the Panel on Cost-effectiveness in Health and Medicine. JAMA 276: 1253–1258. 8849754

[15] World Health Organization (2011) Global price reporting mechanism. Available: http://www.who.int/hiv/amds/gprm/en/. Accessed 22 March 2012.

[16] Government of the Republic of Zambia, Ministry of Health. Medical Stores Limited: 2010 catalogue. Lusaka, Zambia: Government of the Republic of Zambia, Ministry of Health; 2010.

[17] Bratt JH, Torpey K, Kabaso M, Gondwe Y (2011) Costs of HIV/AIDS outpatient services delivered through Zambian public health facilities. Trop Med Int Health 16: 110–118. 20958891 10.1111/j.1365-3156.2010.02640.x

[18] Tagar E, Sundaram M, Condliffe K, Over M, Assefa Y, et al. The cost of scaling-up antiretroviral treatment: a costing study in 161 representative facilities in Ethiopia, Malawi, Rwanda and Zambia [abstract THPE738]; XIX International AIDS Conference, 22–27 July 2012; Washington, DC, USA.

[19] Marseille E, Giganti MJ, Mwango A, Chisembele-Taylor A, Mulenga L, et al (2012) Taking ART to scale: determinants of the cost and cost-effectiveness of antiretroviral therapy in 45 clinical sites in Zambia. PLoS One 7: e51993. 23284843 10.1371/journal.pone.0051993 PMC3527397

